# Protective Role of *Ficus carica* Stem Extract against Hepatic Oxidative Damage Induced by Methanol in Male Wistar Rats

**DOI:** 10.1155/2012/150458

**Published:** 2011-11-17

**Authors:** Mongi Saoudi, Abdelfattah El Feki

**Affiliations:** ^1^Animal Ecophysiology Laboratory, Sciences Faculty of Sfax 3038, Tunisia; ^2^Department of life Sciences, Sciences Faculty of Sfax, BP 1171, Sfax 3000, Tunisia

## Abstract

The present study was aimed to investigate the antioxidant activity of *Ficus carica* stem extract (FE) in methanol-induced hepatotoxicity in male Wistar rats. The rats were divided into two batches: 16 control rats (C) drinking tap water and 16 treated rats drinking *Ficus carica* stem extract for six weeks. Then, each group was divided into two subgroups, and one of them was intraperitoneally injected (i.p.) daily methanol at a dose of 2.37 g/kg body weight i.p. for 30 days, for four weeks. The results showed that FE was found to contain large amounts of polyphenols and carotenoids. The treatment with methanol exhibited a significant increase of serum hepatic biochemical parameters (ALT, AST, ALP, and LDH) and hepatic lipid peroxidation. Hepatic antioxidant enzymes, namely, SOD, CAT, and GSH-Px, were significantly decreased in methanol-treated animals. FE treatment prior to methanol intoxication has significant role in protecting animals from methanol-induced hepatic oxidative damage.

## 1. Introduction

Methanol, also known as methyl alcohol and wood alcohol, is a primary alcohol with the chemical formula CH_3_OH. It is a highly toxic alcohol commonly found in automobile windshield washer solvent, gas line antifreeze, copy machine fluid, fuel for small stoves, and paint strippers and used as industrial solvent. Methanol, the simplest of all alcohols, is toxic to humans. In developing countries like India, methanol is a common adulterant in country liquor which increases the chance of accidental poisoning enormously. The methanol could initiate ROS formation directly via a free radical intermediate, or possibly indirectly through mechanisms like the activation and/or enhancement of ROS-producing NADPH oxidases, which has been reported for ethanol [1]. It is associated with mitochondrial damage and increased microsomal proliferation resulting in increased production of oxygen radicals [2]. 

These factors together with the excess of formaldehyde, formed during acute intoxication, cause significant increases in lipid peroxidation. Products of lipid peroxidation are very harmful to cells ultimately causing their death and can act as second messengers of a complex chain reaction [3].

Liver diseases are among the most serious health problems in the world today, and their prevention and treatment options still remain scarce despite tremendous advances in modern medicine. The pathogenesis of hepatic diseases as well as the role of oxidative stress and inflammation therein is well recognized [4], and consequently, blocking or retarding the chain reactions of oxidation and inflammation development could be promising therapeutic strategies for prevention and treatment of liver injury. Herbs have recently attracted attention as health beneficial foods and as source materials for drug development. Medicinal plant extracts are being increasingly utilized to treat a wide variety of clinical diseases including liver disease [5], ischemia, reperfusion injury, atherosclerosis, acute hypertension, haemorrhagic shock, diabetes mellitus, and cancer [6]. Various species of figs are used in traditional medicine. Figs are a widespread species commonly grown, especially in warm, dry climates. The ideal condition for intensive cultivation of figs is a semi-arid climate with irrigation. The world production of figs is about one million tons, and it is mostly concentrated in the Mediterranean. *Ficus carica* (Family: Moraceae) is commonly referred to as “Fig.” Figs have been traditionally used for their medicinal benefits as metabolic, cardiovascular, respiratory, antispasmodic, and anti-inflammatory remedy [7]. The leaves and the fruits of *Ficus carica* are traditionally used as laxative, stimulant, antitussive, emollient, and resolvent as well as against throat diseases [8, 9]. A decoction prepared from its leaves is used for hemorrhoids, whereas an infusion of its fruits can safely be used as a laxative for children. The fruit is mildly laxative, demulcent, digestive, and pectoral. The leaf decoction is taken as a remedy for diabetes and calcifications in the kidneys and liver [10]. Some recent works have reported that fig antioxidants can protect lipoproteins in plasma from oxidation and produce a significant increase in plasma antioxidant capacity for 4 h after consumption [11]. *F. carica* was chosen for its abundance of phenols, essential oils, and flavonoids, and *F. carica* stem has not been investigated. The aim of this study was undertaken to test the efficacy of aqueous extract prepared from *F. carica* stem against hepatic injury induced by methanol in rats to determine the possible use of this plant in preventing hepatic damage.

## 2. Materials and Methods

### 2.1. Chemicals

2,2-Diphenyl-l-picrylhydrazyl (DPPH), butylated hydroxytoluene (BHT), thiobarbituric acid (TBA), glutathione (GSH), oxidised glutathione (GSSG), glutathione reductase (GR), bovine serum albumin (BSA), and 2,4-dinitrophenyl hydrazine (DNPH) were purchased from Sigma Chemical, France. Trichloroacetic acid (TCA), hydrogen peroxide (H_2_O_2_), 5, 5′ dithiobis (2-nitrobenzoic acid) (DTNB), the Folin-Ciocalteu reagent, sodium carbonate (Na_2_CO_3_), and other solvents were of analytical grade and were freshly prepared in distilled water.

### 2.2. Preparation of *Ficus carica* Stem Extracts (FE)

The *Ficus carica *stems were collected from a culture area located in Kasserine region, Tunisia. Stem samples were ground, put in water, and shaken (10 g/L, v/w) for 15–20 min, and then filtered using the Whatman filter paper. The aqueous extract was given as beverage instead of tap water.

### 2.3. Total Phenolic Content

Total phenolic content was determined using the Folin-Ciocalteu method [12] adapted to a microscale. Briefly, 10 *μ*L diluted extract solution was shaken for 5 min with 50 *μ*L of the Folin-Ciocalteau reagent. Then 150 *μ*L of 20% Na_2_CO_3_ was added and the mixture was shaken once again for 1 min. Finally, the solution was brought up to 790 *μ*L by adding distilled water. After 90 min, the absorbance at 760 nm was evaluated using a spectrophotometer. Gallic acid was used as a standard for the calibration curve. The phenolic content was expressed as mg gallic acid equivalent/gram of dry extract using the linear equation based on the calibration curve.

### 2.4. Determination of Total Flavonoid Content

The flavonoid content in extracts was determined spectrophotometrically according to [13], using a method based on the formation of a flavonoid–aluminium complex, having the maximum absorption at 430 nm. The flavonoid content was expressed in mg of quercetin equivalent per g of dry plant extract (mg QE/g).

### 2.5. Antioxidant Testing Assays

#### 2.5.1. DPPH Radical-Scavenging Activity

Radical-scavenging activity of *F. carica* stem extracts was determined using DPPH as a reagent according to the method of [14] with slight modifications. Briefly, 1 mL of a 4% (w/v) solution of DPPH radical in methanol was mixed with 500 *μ*L of sample solutions in ethanol at different concentrations. The mixture was incubated for 20 min in the dark at room temperature. The scavenging capacity was determined spectrophotometrically by monitoring the decrease in absorbance at 517 nm against a blank using a spectrophotometer (Bio-Rad SmartSpec Plus). Lower absorbance of the reaction mixture indicates higher free-radical-scavenging activity. Ascorbic acid was used as positive control. The percent DPPH scavenging effect was calculated using the following equation: DPPH scavenging effect (%) = (*A*_control_ − *A*_sample_/*A*_control_) × 100, where A_control_ is the absorbance of the control reaction containing all reagents except the tested compound. A_sample_ is the absorbance of the test compound. Extract concentration providing 50% inhibition (IC_50_) was calculated from the graph-plotting inhibition percentage against extract concentration. Tests were carried out in triplicate.

#### 2.5.2. Hydroxyl-Radical-Scavenging Activity

The hydroxyl-radical-scavenging activity was determined according to the colorimetric deoxyribose oxidation by the Fenton reaction leading to malondialdehyde [15]. The hydroxyl radicals were generated from the Fe^3+^/ascorbate/EDTA/H_2_O_2_ system in the non-site-specific assay or Fe^3+^/ascorbate/H_2_O_2_ in the site-specific assay. The reacting mixture of the deoxyribose assay contained in a final volume of 1 mL the following reagents: 200 *μ*L KH_2_PO_4_–KOH (100 mM), 200 *μ*L deoxyribose (15 mM), 200 *μ*L FeCl_3_ (500 *μ*M), 100 *μ*L EDTA (1 mM), 100 *μ*L ascorbic acid (1 mM), 100 *μ*L H_2_O_2 _(10 mM), and 100 *μ*L sample. Reaction mixtures were incubated at 37°C for 1 h. At the end of the incubation period, 1 mL of 1% (w/v) TBA was added to each mixture followed by the addition of 1 mL of 2.8% (w/v) TCA. Solutions were heated on a water bath at 80°C for 20 min to develop the pink-coloured malondialdehyde-thiobarbituric acid, MDA-TBA_2_ adduct, and the absorbance of the resulting solution (total volume = 3.0 mL) was measured at 532 nm. Mannitol, a classical hydroxyl radical scavenger was used as positive control. The inhibition ratio of the extract (%) was calculated using the following formula:

(1) $$\begin{array}{cc} \text{Inhibition}\;\text{ratio}\;\left(\text{\%}\right) & =\left(\frac{A_{\text{control}\;532\;\text{nm}}-A_{\text{sample}\;532\;\text{nm}}}{A_{\text{control}\;532\;\text{nm}}}\right)\times 100. \end{array}$$

### 2.6. Animals

Adult male albino *Wistar* rats weighing 180 to 200 g were obtained from the Central Pharmacy of Tunisia (SIPHAT, Tunisia). The animals were quarantined and allowed to acclimatize for a week prior to experimentation. The animals were handled under standard laboratory conditions of a 12 h light/dark cycle in a temperature- and humidity-controlled room. Food and water were available *ad libitum*. Our Institutional Animal Care and Use Committee approved the protocols for the animal study, and the animals were cared for in accordance with the institutional ethical guidelines.

### 2.7. Treatment

After acclimatization, the rats were divided into two batches: 16 control rats (C) drinking tap water and 16 treated rats drinking *Ficus carica *stem extract (FE) for six weeks. Then, each group was divided into two subgroups, and one of them was intraperitoneally injected (i.p.) daily, for four weeks, with methanol (2.37 g/kg b.wt.) according to [16]. After treatment, 8 rats of each group were sacrificed by cervical dislocation under anaesthesia by i.p. injection of chloral hydrate; livers were excised, washed and rinsed with ice-cold saline, and homogenized (Ultra-Turrax T25, Germany) (1 : 2, w/v) in 50 mmol L^−1^ phosphate buffer (pH 7.4). The homogenate and supernatant were frozen at −30°C in aliquots until used for antioxidant enzymes and lipid peroxidation. The protein content of the supernatant was determined using the method of [17].

### 2.8. Serum Parameters

Serum samples were obtained by the centrifugation of blood at 4000 rpm for 15 min at 4°C and were then divided into Eppendorf tubes. Isolated sera were stored at −30°C until they were used for the analyses. The levels of serum alanine aminotransferase (ALT), aspartate aminotransferase (AST), alkaline phosphatase (ALP), and lactate dehydrogenase (LDH) were measured using commercial kits according to the manufacturer's directions.

### 2.9. Oxidative Stress Analysis

#### 2.9.1. Thiobarbituric Acid Reactive Substance (TBARS) Measurements

Lipid peroxidation in the tissue homogenate was estimated by measuring thiobarbituric acid reactive substances (TBARS) and was expressed in terms of malondialdehyde (MDA) content which is the end product of lipid peroxidation, according to [18]. In brief, 125 *μ*L of supernatants were homogenized by sonication with 50 *μ*L of TBS, 125 *μ*L of TCA-BHT in order to precipitate proteins and centrifuged (1000 g, 10 min, 4°C). 200 *μ*L of obtained supernatant were mixed with 40 *μ*L of HCl (0.6 M) and 160 *μ*L of TBA dissolved in Tris, and the mixture was heated at 80°C for 10 min. The absorbance of the resultant supernatant was read at 530 nm. The amount of TBARS was calculated by using an extinction coefficient of 156 × 10^5^ mM^−1^ cm^−1^.

#### 2.9.2. Antioxidant Enzyme Studies

In liver tissues, SOD activity was determined according to the colorimetric method of [19] using the oxidizing reaction of nitroblue tetrazolium (NBT); CAT activity was measured by the UV colorimetric method of [20] using H_2_O_2_ as substrate; glutathione peroxidase (GSH-Px) activity was measured by a modification of the colorimetric method of [21] using H_2_O_2_ as substrate and the reduced GSH.

### 2.10. Statistical Analysis

All values are expressed as mean ± S.E.M. The results were analyzed by one-way analysis of variance (ANOVA) followed by Tukey test for multiple comparisons using SPSS for Windows (version 11). Differences were considered significant when  *P* < 0.05.

## 3. Results

### 3.1. Total Phenolics (TPCs) and Flavonoids

Total phenolics and flavonoids are shown in Table 1. The *F. carica* stem extract contained phenolic compounds (133 ± 3.50 mg GAE/g), in which its level was expressed as gallic acid equivalents. Total flavonoids were expressed as quercetin equivalent per g of dry plant extract. The *F. carica* stem extract contained flavonoid compounds (43.25 ± 2.0 mg QE/g).

### 3.2. Antioxidant Capacities of *F. carica* Stem Extract

**Figure 1:**
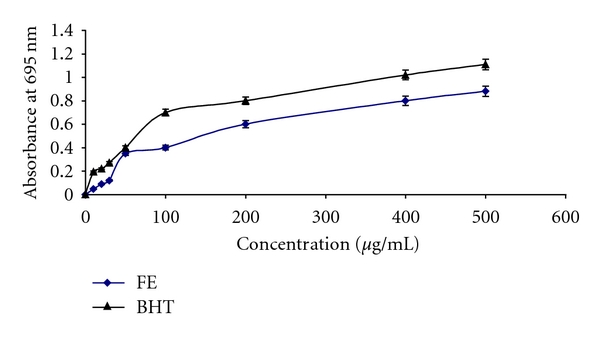
DPPH-radical-scavenging activity of *Ficus carica* stem extract and positive control BHT at different concentrations. Data represent the means ± SEM (*n* = 3).

The antioxidant activity of *F. carica* stem extract was determined by its capacity to scavenge the stable free radicals DPPH, which has been widely used to test free-radical-scavenging activity, and the results were compared with the scavenging ability of control samples of BHT (Figure 1). In the DPPH scavenging assay, the IC50 (the concentration required to scavenge 50% of radical) value of FE was 420 ± 3.30 *μ*g/mL. In the *β*-carotene-bleaching method, the degree of linoleic acid oxidation is determined by measuring oxidation products (lipid hydroperoxides, conjugated dienes, and volatile by-products) of linoleic acid which simultaneously attack *β*-carotene, resulting in bleaching of its characteristic yellow color in aqueous solution. The antioxidant activity of *F. carica* was evaluated using different concentrations of extracts and was compared with BHT used as reference (Table 2). The addition of the FE fraction and the BHT at a concentration of 800 and 1000 *μ*g/mL prevented the bleaching of *β*-carotene with different degrees. As shown in Figure 1, the FE fraction exhibited high hydroxyl-radical-scavenging activity with an IC_50_ of 420 ± 3.30 *μ*g/mL.

### 3.3. Serum Biochemical Parameters

The effect of aqueous extract of *F. carica* stem on serum transaminases, alkaline phosphatise, and lactate dehydrogenase levels in methanol intoxicated rats is summarized in Table 3. There was a significant increase in serum AST, ALT, ALP, and LDH levels. However, no significant change was observed in FE-treated rats, as well as in FE+M rats showing the protective effects of *F. carica* stem extract against changes induced by methanol treatment. The present study showed that the administration of *F. carica* stem extract reverts the enzyme activities near to normal status.

### 3.4. Effect on Lipid Peroxidation

**Figure 2:**
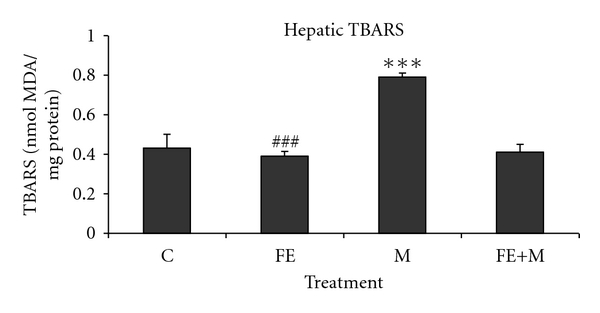
Hepatic TBARS of control and rats treated with methanol (M), *Ficus carica* stem extract (FE), and their combinations (FE + M). Significant differences: values are mean ± SEM for eight rats in each group. FE, M, and FE + M treated groups versus control group; ***P < 0.001, M group versus (FE + M) group; ^###^ *P* < 0.001.

Lipid peroxidation was significantly increased in the animals exposed to methanol as compared with respective controls (Figure 2). The increase in lipid peroxidation induced by methanol was attenuated in *F. carica* stem extract pretreated animals.

### 3.5. Effect on Antioxidant Enzymes

**Figure 3:**
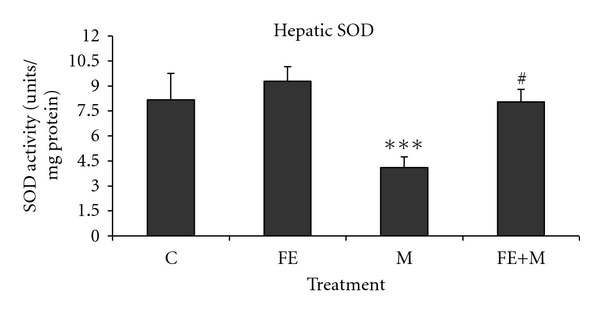
Hepatic SOD of control and rats treated with methanol (M), *Ficus carica* stem extract (FE), and their combinations (FE + M). Significant differences: values are mean ± SEM for eight rats in each group. FE, M, and FE + M treated groups versus control group; ***P < 0.001, M group versus (FE + M) group; ^#^ *P* < 0.05.

**Figure 4:**
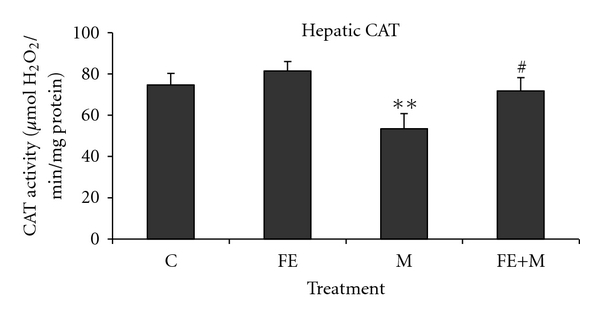
Hepatic CAT of control and rats treated with methanol (M), *Ficus carica* stem extract (FE), and their combinations (FE + M). Significant differences: values are mean ± SEM for eight rats in each group. FE, M, and FE + M treated groups versus control group; **P < 0.01, M group versus (FE + M) group; ^#^ *P* < 0.05.

**Figure 5:**
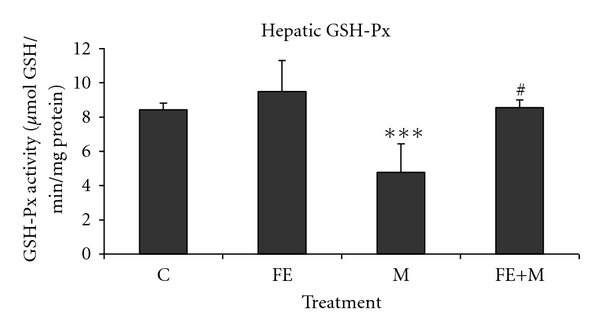
Hepatic GSH-Px of control and rats treated with methanol (M), *Ficus carica* stem extract (FE), and their combinations (FE + M). Significant differences: values are mean ± SEM for eight rats in each group. FE-, M- and FE + M-treated groups versus control group; ***P < 0.001, M group versus (FE + M) group; ^#^ *P* < 0.05.

Hepatic superoxide dismutase, catalase, and glutathione peroxidase activities were decreased in methanol-treated group as compared with respective controls. The animals pre-treated with *F. carica* stem extract exhibited significant restoration in SOD (Figure 3), CAT (Figure 4), and GSH-Px (Figure 5) activities. No significant change was observed in FE-treated rats, as well as in FE+M rats showing the protective effects of *F. carica* stem extract against changes induced by methanol treatment.

## 4. Discussion

In our study, the phenolic estimation reveals that aqueous extract of *F. carica* stem extract contains considerable amount of polyphenolic and flavonoid compounds. The antioxidant activity of the plant extracts and standard was assessed on the basis of the radical-scavenging effect on the DPPH^•^ free radical. A large variety of in vitro methods assessed radical-scavenging ability of certain agents from natural and synthetic source. DPPH^•^ free radical has been used to assess the ability of phenolic compounds to transfer labile hydrogen atoms to radicals [22]. Total H atom donating capacities are evaluated in the IC50 index, which is defined as the concentration of antioxidants needed to scavenge 50% of DPPH^+^. Our results showed capacity of FE had effective DPPH^•^ (420 ± 3.30 *μ*g/mL) scavenging activity in a concentration dependant. Several polyphenols including flavonoids are found in figs [23]. On the other hand, previous works demonstrated that *F. carica* was chosen for its abundance of phenols, essential oils, and flavonoids, which are effective on bacteria through compounds produced such as resveratrol, psoralen, and bergapten. These compounds have demonstrated antibacterial activity and could be used commercially as a means of phytopathogenic bacteria control [24, 25]. The studies of [26] denoted that phytochemical analysis revealed that the aqueous extract of ripe-dried fruit of *F. carica* contains alkaloids, flavonoids, coumarins, saponins, and terpenes. The extract was tested for its possible spasmolytic effect on isolated rabbit jejunum preparations, where it inhibited the contractions at the concentration, thus showing the antispasmodic effect. The relaxant effect was dose dependent with an IC50 value of 0.74 mg/mL. The observed antispasmodic effect of *F. carica* was reversible returning to normal spontaneous contractions within 2–4 min of washing the tissue with fresh bathing physiological solution [26–28]. It can be important that the content of polyphenolic compounds of *F. carica* extract could be responsible for the radical-scavenging activity in methanol toxicity. Liver is a major site for metabolism of exogenous chemicals, resulting in the formation of metabolites which may be more or less toxic than the parent compound. The aqueous extract of *F. carica* stem, administered prophylactically, exhibited significant protection against methanol-induced liver injury as manifested by the reduction in toxin-mediated rise in serum transaminases, ALP, and LDH in rats. The results of [29] are similar to those mentioned in our study which demonstrated that the extract of a medicinal plant *Oldenlandia umbellata* administered orally to rats reversed the altered hepatic serum marker enzymes (AST, ALT, and ALP) by CCl4 to almost normal. The rise in serum levels of AST, ALT, and ALP has been attributed to the damaged structural integrity of the liver, because they are cytoplasmic in location and released into circulation after cellular damages [30]. Our results are in agreement with those of [31] which mentioned that the oral administration of *Coriandrum sativum* at a dose of 200 mg/kg body weight to rats returned the activities of serum AST and ALT as a hepatic biomarker to normal values, which may be a consequence of the stabilization of plasma membrane as well as repair of hepatic tissue damage caused by CCl4. Lactate dehydrogenase is a well-known marker for cell toxicity. When cells are damaged due to oxidative stress or destroyed due to deficient oxygen or glucose supply, the membrane becomes permeable or may rupture which results in the leakage of this enzyme. This enzyme enters into the blood stream thus increasing its concentration in the serum [32]. In the present study, LDH leakage is significantly increased in the serum of rats exposed to methanol. FE extract administration to methanol-treated rats significantly decreased LDH level in serum compared to rats treated with methanol alone. Further, the results of our study showed that coadministration of *F. carica* extract diminished methanol-induced oxidative stress by increasing antioxidant status. These results agree with the hypothesis that oxidative damage is neutralized when antioxidants are administered before or after the induction of oxidative stress [33]. Faremi et al. [34] recently revealed that *Phyllanthus amarus *(*P. amarus*) leaf extract had a hepatoprotective role against ethanol-induced oxidative damage in adult male *Wistar* rats. *P. amarus* cotreatment significantly reduced the activities of plasma AST, ALT, and ALP in the ethanol-challenged rats. This investigator had reported similar observations to our study in the levels of plasma AST, ALT, and ALP activities. In the present study, methanol caused an increase in LPO level in liver tissues which is an indicator of oxidative stress. Furthermore, LPO products are measured as index of oxygen free radical. The present result of increased LPO level after methanol exposure indicates an increased oxygen free radical generation by alcohols. This result is in accordance with previous studies using methanol and other alcohols [35]. FE administration to methanol-treated rats resulted in a significant decrease in LPO level compared to rats treated with methanol alone. These results are similar to the observation of another study where figs extract was shown to decrease LPO level in streptozotocin-induced damage in albino rats [36]. Recent studies [37] have provided evidence that aqueous extract of *Taraxacum officinale* root, a herbal medicine, exhibited significant decrease in hepatic lipid peroxidation against liver damage mediated by alcohol in vivo. Compared with the control group, the methanol-intoxicated animals exhibited a significant decrease in SOD, CAT, and GPx levels. These changes were significantly reversed upon treatment with FE. Therefore, some polyphenols and flavonoids exert a stimulatory action on transcription and gene expression of certain enzymes. The antioxidant enzyme system plays an important role in the defence of cells against oxidative insults. Both SOD and CAT are key antioxidant enzymes that protect against oxidative stress and tissue damage [38]. These enzymes are critical for defence mechanisms against the harmful effects of reactive oxygen species (ROS) and free radicals in biological systems. The SOD converts superoxide radicals (O^•^_2_) into H_2_O_2_ and O_2_, thus participating with other antioxidant enzymes in the enzymatic defense against oxygen toxicity. CAT is a key component of the antioxidant defense system. Inhibition of this protective mechanism results in enhanced sensitivity to free-radical-induced cellular damage. The decrease of CAT may result in a lot of deleterious effects due to the accumulation of superoxide radicals and hydrogen peroxide [39]. GPx content was another important parameter that revealed oxidative damage in liver. Reduction in liver GPx activity in methanol-treated rats as observed in this study indicated the damage in liver. The fact that FE treatment reduced elevated LPO and increased levels of SOD, CAT, and GPx indicated that FE may prevent the peroxidation of lipids by methanol. Our study showed that total phenolics were important antioxidants in *F. carica* stem. Moreover, these findings supported the beneficial effect of *F. carica* in maintaining the hepatocytes integrity and function. It is conceivable that these effects may be due, at least in part, to its antioxidant activity. The antioxidant enzyme (SOD, CAT, and GPx) activities were increased by FE in liver tissues when compared with those administered with methanol only. These results are in accordance with those of Srivastava and Shivanandappa [40] which demonstrated that the aqueous extract of the roots of *Decalepis hamiltonii*, a climbing shrub that grows in the forests of peninsular India and is consumed for its health-promoting properties, prevented CCl4-induced oxidative stress in the rat liver by inhibiting lipid peroxidation and protein carbonylation, restoring the levels of antioxidant enzymes (SOD, CAT, GPx, GR, and GST) and glutathione. The concentration of LPO decreased significantly following FE administration in the liver tissues. These results clearly show that the antioxidant property of FE reduces significantly the damage of methanol-induced liver injury and activates the biological defense system of the liver. Furthermore, FE appears to scavenge free radicals [41], as shown by the inhibition of lipid peroxidation induced by methanol in vivo.

## 5. Conclusion

In the present study, the stem extracts of *F. carica* have been demonstrated to possess excellent antioxidant activities by various in vitro and in vivo assays. The various in vitro antioxidant tests proved that the plant possesses components such as polyphenols and flavonoids with scavengers of free radicals. Current study also demonstrates that *F. carica* aqueous extract could reduce methanol-induced toxicity, particularly hepatotoxicity, by suppressing alanine aminotransferase (ALT), aspartate aminotransferase (AST), alkaline phosphatase (ALP), and lactate dehydrogenase (LDH) activities, inhibiting lipid peroxidation and increasing antioxidant enzyme activities. The observed strong antioxidant activity of *F. carica* may be due to the presence of several phenolic compounds, such as flavonoids. Further investigation is needed to isolate and identify the active principle involved in antioxidant activities of this plant.

## Figures and Tables

**Table 1:** Amounts of total flavonoid and total phenolic compounds and evaluation of the IC_50_ values of the DPPH free-radical-scavenging assay of *Ficus carica *stem extract. BHT was used as standard. Each value represents the mean ± SEM of three experiments.

Extract/fraction	Phenolic content mg GAE/g	Flavonoid content mg QE/g	DPPH IC_50_ (*μ*g/mL)
FE	133 ± 3.50	43.25 ± 2.0	420 ± 3.30

(mg GAE /g): mg of gallic acid equivalent per g of dry plant extract.

(mg QE/g): mg of quercetin equivalent per g of dry plant extract.

IC_50 _(*μ*g/mL) the IC_50_ values corresponding to the amount of extract required to scavenge 50% of radicals present in the reaction mixture.

**Table 2:** Inhibition of lipid peroxidation obtained by *Ficus carica* stem extract as assessed by the coupled oxidation of *β*-carotene and linoleic acid over 120 min. Data represent the means ± SEM (*n* = 3).

Concentration (*μ*g/mL)	% inhibition of lipid peroxidation in FE extract
100	52.70 ± 0.46
200	58.0 ± 1.25
400	62.90 ± 1.30
800	75.80 ± 1.50
1000	80.0 ± 1.08

**Table 3:** Hepatic markers in the serum of control and rats treated with methanol (M), *Ficus carica* stem extract (FE), and their combinations (FE + M).

Parameters	Experimental groups			
	Control	FE	M	FE + M
AST (U/l)	179.6 ± 11.8	181 ± 9.8	195.5 ± 7.8***	177.2 ± 12.4^# #^
ALT (U/l)	45.3 ± 5.7	48 ± 7.8	66.3 ± 9.4**	42.2 ± 4.7^#^
ALP (U/l)	65.8 ± 4.33	67 ± 2.9	81.5 ± 9.67**	69.5 ± 8.2^#^
LDH (U/l)	833.45 ± 55.33	923.66 ± 90.47	1155.54 ± 74.67***	919.63 ± 88.72^#^

AST: aspartate aminotransferase; ALT: alanine aminotransferase; ALP: alkaline phosphatase; LDH: lacatate dehydrogenase.

Significant differences: values are mean ± SEM for eight rats in each group. FE-, M- and FE + M-treated groups versus control group; *P < 0.05, **P < 0.01, ***P < 0.001, M group versus (FE + M) group; ^#^ *P* < 0.05, ^# #^ *P* < 0.01.
